# Global research priorities for infections that affect the nervous system

**DOI:** 10.1038/nature16033

**Published:** 2015-11-19

**Authors:** Chandy C. John, Hélène Carabin, Silvia M. Montano, Paul Bangirana, Joseph R. Zunt, Phillip K. Peterson

**Affiliations:** 1 Ryan White Center for Pediatric Infectious Disease and Global Health, Department of Pediatrics, Indiana University School of Medicine, Indianapolis, Indiana 46202, USA.; 2 Department of Biostatistics and Epidemiology, College of Public Health, University of Oklahoma Health Sciences Center, Oklahoma City, Oklahoma 73104, USA.; 3 Department of Bacteriology, US Naval Medical Research Unit No. 6, Lima, Peru.; 4 Department of Psychiatry, Makerere University College of Health Sciences, Kampala, Uganda.; 5 Department of Epidemiology, University of Washington, Seattle, Washington 98195, USA.; 6 Division of Infectious Diseases and International Medicine, University of Minnesota, Minneapolis, Minnesota 55455, USA.

## Abstract

Infections that cause significant nervous system morbidity globally include viral (for example, HIV, rabies, Japanese encephalitis virus, herpes simplex virus, varicella zoster virus, cytomegalovirus, dengue virus and chikungunya virus), bacterial (for example, tuberculosis, syphilis, bacterial meningitis and sepsis), fungal (for example, cryptococcal meningitis) and parasitic (for example, malaria, neurocysticercosis, neuroschistosomiasis and soil-transmitted helminths) infections. The neurological, cognitive, behavioural or mental health problems caused by the infections probably affect millions of children and adults in low- and middle-income countries. However, precise estimates of morbidity are lacking for most infections, and there is limited information on the pathogenesis of nervous system injury in these infections. Key research priorities for infection-related nervous system morbidity include accurate estimates of disease burden; point-of-care assays for infection diagnosis; improved tools for the assessment of neurological, cognitive and mental health impairment; vaccines and other interventions for preventing infections; improved understanding of the pathogenesis of nervous system disease in these infections; more effective methods to treat and prevent nervous system sequelae; operations research to implement known effective interventions; and improved methods of rehabilitation. Research in these areas, accompanied by efforts to implement promising technologies and therapies, could substantially decrease the morbidity and mortality of infections affecting the nervous system in low- and middle-income countries.

Recent improvements in the detection of infectious organisms that can affect the nervous system has led to the realization that a substantial proportion of chronic neurological, cognitive and behavioural disease may actually have an acute and preventable origin. Infectious organisms may infect the nervous system directly, as in rabies and bacterial meningitis, or may cause neurocognitive disorders in the absence of direct infection of the nervous system, as in malaria or hookworm infection. This Review identifies global research priorities for infections that affect the nervous system, with the ultimate goal of stimulating research in these priority areas to substantially reduce morbidity associated with nervous system infections worldwide.

## METHODS

For this Review, we chose illustrative infections that cause considerable nervous system morbidity in children and adults in low- and middle-income countries (LMICs). These infections are examples and are not meant to be exhaustive. Estimates of infection global frequency and types of nervous system involvement were obtained through PubMed searches using the infection name and were accompanied by any of the following terms: neurologic, nervous system, cognition, cognitive, development, neurodevelopment, impairment, deficit, sequelae, brain injury, brain damage, mental health, behavioral or neuropathy. If available, World Health Organization (WHO) documents were also reviewed for each disease. The authors came to a consensus on the key research priority areas, on the basis of a literature review and research experience.

## INFECTIONS AFFECTING THE NERVOUS SYSTEM

The global distribution, frequency and types of neurological, cognitive and mental health disorders associated with key infections are presented in Table 1. For classification purposes, infections are reviewed according to type of microorganism (virus, bacteria, fungus or parasite) in the sections that follow. However, microorganisms within a group (for example, the viruses HIV and rabies) can affect the nervous system in as varied a manner as microorganisms of different groups (for example, the virus HIV and the malaria-causing parasite *Plasmodium falciparum*).

### Viral infections

Worldwide, rabies and Japanese encephalitis virus (JEV) are responsible for an estimated annual mortality of 60,000 and 17,000 people, respectively^1,2^. Cases of JEV encephalitis are restricted to Asia, whereas rabies is a scourge throughout Southeast Asia, Africa and Latin America and occurs, although less frequently, in other areas worldwide. Rabies and herpes simplex virus (HSV) encephalitis, which is also present worldwide, lead to high mortalities without treatment^3^. JEV has variable mortality, depending, in part, on the infected individual's age. Among survivors, long-term cognitive or neurological impairment is present in as many as 70% of those with HSV encephalitis^4^ and 30–50% of those with JEV encephalitis^5^. Most cases of JEV infection (as opposed to encephalitis) are asymptomatic or mildly symptomatic and require no treatment^6^, but the long-term neurocognitive consequences of asymptomatic or mildly symptomatic JEV infections are unknown.

Varicella zoster virus (VZV), the cause of chickenpox, can, like other herpesviruses, establish a latent infection. Reactivation is usually owing to suppression of cell-mediated immunity, most commonly age-related immunosenescence. Central nervous system (CNS) reactivation is relatively uncommon, but reactivation in a dorsal root ganglion can lead to herpes zoster, which is associated with debilitating chronic pain^7^. Herpes zoster is the single most common infection of the nervous system, with an estimated one million new cases each year in the United States alone^8^. There is little data on nervous system VZV infection in LMICs.

Congenital cytomegalovirus (CMV) infection is the most common acquired cause of hearing loss in children in the United States^9^. The percentage of the population testing positive for CMV is higher in LMICs than in high-income countries, but the incidence of congenital CMV infection and of symptomatic CNS disease and hearing loss in most LMICs remains almost unknown^10^.

Dengue, and to a lesser extent chikungunya, viruses will probably become leading global causes of arboviral encephalitis in the next decade^2,11,12^. Although encephalitis is present in only a fraction of those infected with dengue virus, the large number of dengue infections worldwide could lead to hundreds of thousands of encephalitis cases (Table 1).

### HIV and opportunistic infections

In 2012, an estimated 2.3 million people were infected with HIV, and 1.6 million died of AIDS-related illnesses worldwide^13^. HIV-associated neurological syndromes are classified as primary HIV infection, secondary or opportunistic infection, and treatment-related neurological disease. Most often, primary HIV infection causes acute aseptic (viral) meningitis or meningoencephalitis (MEC). HIV-associated neurocognitive disorder (HAND) is a neurodegenerative condition characterized by cognitive, motor and behavioural abnormalities that is becoming more common with an increase of HIV in people older than 50 years^14^. In LMICs, where only one third of patients requiring highly active antiretroviral therapy (HAART) receive it, the opportunistic infections cryptococcal meningitis, tuberculous meningitis, cerebral toxoplasmosis, progressive multifocal leukoencephalopathy and CNS cytomegalovirus infection remain common^15^.

The widespread implementation of combination antiretroviral therapy (cART) has changed the presentation, manifestation and epidemiology of many conditions and opportunistic infections, owing to immune reconstitution syndrome and increased prevalence of cognitive impairment and neuropathy with additional morbidities in patients who are now living longer^16^. The epidemiology and neurological outcomes of HIV infection are also affected by underlying malnutrition and variations in endemic pathogens.

Cryptococcal meningitis is a leading cause of mortality in LMICs where access to antiretroviral therapy is limited^17^. Most cases occur in sub-Saharan Africa, followed by South and Southeast Asia^18^. Seroprevalence for *Toxoplasma gondii* in people with HIV ranges from 10% to 80% with the highest proportions in African countries^19^, and cerebral toxoplasmosis is the most common cerebral mass lesion in patients with AIDS.

### Bacterial infections

The most common bacterial infections affecting the nervous system are sepsis and meningitis in neonates; bacterial meningitis due to *Streptococcus pneumoniae*, *Haemophilus influenzae* type b and *Neisseria meningitidis* in children and adults; and tuberculous meningitis in children and adults.

Neonatal meningitis and neonatal sepsis are associated with long-term neurological and cognitive impairment^20^; primarily impairment of hearing, vision or motor function; cerebral palsy; and epilepsy. In LMICs, it is estimated that 23% of neonates who have survived meningitis sustain moderate to severe neurodevelopmental impairment^21^. *Staphylococcus aureus*, Gram-negative infections, including *Escherichia coli*, *Klebsiella pneumoniae*, *Acinetobacter,* non-typhoidal *Salmonella* and group B *Streptococcus*, are the leading causes of neonatal sepsis and meningitis in most LMICs^22–24^. Recent reports from Africa and India suggest an alarming increase in drug resistance among Gram-negative organisms infecting neonates^24,25^.

In children and adults in high-income countries, bacterial meningitis due to *S. pneumoniae*, *H. influenzae* type b and *N. meningitidis* has decreased dramatically following immunization with conjugate vaccines^26^. However, availability of these vaccines in LMICs is variable, and bacterial meningitis still affects 1.2 million individuals annually^26^, causing neurocognitive sequelae in 23% of affected children. Steroids as an adjunctive therapy have reduced neurological sequelae in high-income countries, particularly in adults, but have shown no benefit in LMICs^27^. Factors such as organism strain, co-infections, adjunctive and supportive therapies, and underlying conditions such as poor nutrition that can affect immune response often differ between high-income countries and LMICs, and may play a part in the different results seen in some clinical trials.

Tuberculous meningitis (TBM) occurs in around 1% of all cases of tuberculosis, but is the most severe form of extrapulmonary tuberculosis, resulting in death or severe disability in about 50% of those with the disease^28^. Bacterial meningitis, and particularly TBM, can result in hydrocephalus, which is often difficult to treat in LMICs because neurosurgeons are typically not available and the supplies for ventriculoperitoneal shunt placement are often not present or very limited.

The WHO estimated that approximately 10.6 million new cases of syphilis occurred in 2008 (ref. 29), but precise estimates of the incidence of neurosyphilis are not available.

### Parasitic infections

Malaria in humans is caused by one of five *Plasmodium* species, but neurological disabilities are most frequently associated with *Plasmodium falciparum*. Although *P. falciparum* does not directly infect brain tissue, severe infection can lead to coma. One in four children with cerebral malaria develops long-term cognitive impairment^30^, and recent studies suggest that children with severe malarial anaemia also have long-term cognitive impairment^31^. Behavioural problems and epilepsy are other long-term consequences of cerebral malaria^32^. Children with repeated episodes of uncomplicated malaria have motor and cognitive problems^33^. The mechanisms by which malaria leads to neurocognitive problems are not fully defined, and the neurocognitive burden of malaria owing to other *Plasmodium* species has not been characterized.

Neurocysticercosis, which is endemic in areas with poor pig management practices and sanitation, occurs when the larval stages of *Taenia solium* infect the brain. In LMICs in which neurocysticercosis is endemic, it is the leading identified cause of seizures. The proportion of larval infections migrating to the brain is unknown, but some individuals with neurocysticercosis never show neurological symptoms^34^. Neurocysticercosis can be fatal, most often following complications of surgery to treat the hydrocephalus associated with intraventricular or subarachnoid neurocysticercosis, but overall mortality estimates have been difficult to obtain^35^.

Neuroschistosomiasis results from the migration of schistosome eggs or worms to the brain or spinal cord, and may occur following infection with *Schistosoma japonicum*, *Schistosoma mansoni* or *Schistosoma haematobium*. Brain involvement may occur in the acute phase (acute schistosomal encephalopathy) or in the chronic phase (cerebral schistosomiasis or pseudotumoral encephalic schistosomiasis). The spinal cord may also be involved, often leading to hemiparesis^36^. CNS symptoms and epilepsy are reported to occur in 2.6% and 2.1% of *S. japonicum* infections, respectively^37^. Neuroschistosomiasis can be fatal, especially in its tumour-like form when it affects the cerebellum, but accurate mortality rates are unavailable^38^.

Soil-transmitted helminths (STH) affect millions, most frequently children. Determining the part played by STH in cognitive impairment of children is complicated because STH infection is associated with many confounders, but data that support a role for STH in neurobehavioural outcomes include a study showing that infants and children under 5 years of age with anaemia and STH infection show disturbed social and emotional behaviour. Another study showed that treating school-aged children with antiparasitic drugs and iron supplementation improved attention, memory and processing speed^39^.

## GLOBAL RESEARCH PRIORITIES

Prevention of infections that affect the nervous system is the highest research priority, as complete prevention of infection removes all risk of nervous system sequelae. However, treatment of nervous system sequelae and rehabilitation of individuals with nervous system morbidity are also important for the millions who currently live with the nervous system effects of infections. Prevention and treatment of infections that affect the nervous system requires the identification of the pathogens responsible, the pathogen reservoirs and the potential points at which the pathogen life cycle can be interrupted. Table 1 lists specific pathogens, their known nervous system manifestations, and current knowledge gaps regarding incidence and long-term sequelae for each pathogen. Table 2 outlines whether specific interventions (vaccines, control of zoonotic reservoirs or vector populations, and treatment) are available for each pathogen. Table 3 provides a summary of global research priorities for infections that affect the nervous system. These research priorities are discussed in more detail below.

### Diagnosis

Improved diagnosis lies at the heart of all research priorities for infections that affect the nervous system because all other research areas depend on accurate infection diagnosis. Improved diagnosis requires better tests to detect infection, better clinical diagnostic algorithms to detect infection and better tools to assess the nervous system effects of infection, including cognitive and mental health sequelae.

Affordable, easy-to-use, rapid diagnostic assays — preferably point-of-care — that can identify infections affecting the nervous system are a high priority. This includes diagnostic tests for infections that directly infect nerve cells and those that do not (for example, malaria and STH). For infections that affect the CNS, field diagnoses are needed to identify when the infection has entered the CNS. Serological assays are available to detect schistosomiasis^36^ and cysticercosis, but these tests are not specific for CNS infection, and the blood–brain barrier may prevent the detection of antigens in serum^40^. In the case of bacterial, fungal or viral CNS infections, although lumbar puncture to obtain cerebrospinal fluid is a routine procedure at many centres in LMICs, most lack the capacity for standard bacterial, fungal or viral cultures, let alone more sophisticated testing such as PCR, which is essential for detecting many viral infections. Even in high-income countries with advanced molecular diagnostics, an aetiological agent is identified in less than half of individuals with encephalitis. Because many cases of idiopathic encephalitis are probably caused by viruses still to be characterized, the development of metagenomic and high-throughput screening techniques for viral detection is a research priority, with the goal of eventually developing low-cost diagnostic point-of-care assays for the pathogens identified. For certain CNS infections, notably neurocysticercosis, neuroschistosomiasis, CNS tuberculosis and CNS toxoplasma infection, neuroimaging with CT scans or MRI is needed to make a diagnosis. Although availability of neuroimaging is becoming more widespread, many facilities in LMICs still lack these costly imaging modalities, underscoring the need for research on simple, accurate, low-cost, point-of-care diagnostic tests for detecting infections that affect the nervous system.

One example of how improved diagnostic tools can have an impact is a study^41^ from India in which a simple diagnostic algorithm and basic treatment for neonatal sepsis, all performed by village health workers, led to a 63% reduction in neonatal mortality among preterm infants^41^. Simple algorithms for other infections that affect the nervous system, coupled with the ability to provide effective therapy following diagnosis, or appropriate referral for screening algorithms, have the potential to substantially reduce morbidity and possibly mortality from these infections. Improved cross-cultural measurements of neurodevelopment and mental health are a key research priority, and reviewed in the article in this collection on child neurodevelopment (see page S155).

### Epidemiology and primary prevention

The lack of affordable, non-invasive, rapid diagnostics for infection and nervous system effects of infection limits our ability to quantify the burden of infection-related nervous system disability (Table 1). Well-designed studies of disease epidemiology are also required for the accurate measurement of disease incidence, and of the type and duration of nervous system sequelae of infection.

A challenge to the estimation of infection-related nervous system disease is that the symptoms these infections result in, such as epilepsy, hemiparesis or cognitive impairment, are included as ‘chronic diseases’ in global burden estimates. Careful epidemiological assessment could lead to the more accurate attribution of a portion of ‘chronic disease’ to its infectious component. For example, the *Global Burden of Disease Study 2010* (ref. 42) attributed some of the disability-adjusted life years of epilepsy to neurocysticercosis. This infection is also associated with stroke^43^, which was ranked third in terms of disability-adjusted life years in 2010 (ref. 42), but none of this burden was attributed to neurocysticercosis owing to lack of data. Thus the true burden of nervous system disease owing to neurocysticercosis was probably significantly underestimated.

The most cost-effective method of preventing infection is immunization, discussed in the section on vaccines below. For infections for which there is no immunization, or for which immunization is not highly successful, research is required on sustainable preventive methods. For vector-borne illness, for example, insecticide-based interventions such as insecticide-treated bed nets have reduced malaria incidence and mortality in many areas^44^. But increasing pyrethroid resistance^45^ highlights the need for ongoing research even for interventions with documented past success.

### Pathogenesis

Disease pathogenesis may be the most neglected research focus of infection-related nervous system disease in LMICs. Although some studies on the pathogenesis of infection-related nervous system disease in individuals in high-income countries are available^46,47^, far fewer studies of pathogenesis have been conducted in individuals from LMICs. Even in high-income countries, studies of infection pathogenesis often use animal models, which may incompletely recapitulate the host response in humans. The host immune response probably contributes to both defence against invading pathogens and subsequent damage to the nervous system^48^, but the type and role of specific cells in the immune response at different infection stages are poorly described. Similarly, it is often unclear which antigens or components of the infecting organism confer neurovirulence. The roles of innate immunity, the microbiome, and co-infection with endemic pathogens, including HIV, in contributing to infection-related nervous system disease are also poorly understood (Box 1). Without an understanding of the pathogenesis of infection-related nervous system disease, it is difficult to rationally plan for adjunctive interventions to prevent or reduce nervous system injury. Although adjunctive interventions have been elusive, those proven successful (for example, steroid treatment in tuberculous meningitis^49^) have made a major difference in improving neurocognitive and behavioural outcomes. An understanding of the development and types of protective immune responses to antigens or antigenic variants of a pathogen is also fundamental to the development of vaccines, which are, in most cases, the most cost-effective method of preventing infection.

### Vaccine development

Vaccines are available to prevent the neurological complications of measles, mumps, rubella, poliomyelitis and varicella virus as well as *H. influenzae* type b, *S. pneumoniae* and *N. meningiditis*. Effective vaccines are also available for rabies and Japanese encephalitis. Together, these vaccines have saved millions of lives as well as prevented long-term nervous system complications in millions of children and adults.

Research priorities for vaccine development include the utilization of disease immunology, epidemiology and pathogenesis studies to develop safe, effective vaccines, and the performance of phase I, II and III trials to determine vaccine efficacy and safety in humans. In *P. falciparum* malaria, for example, knowledge of antibody and T-cell immune responses to circumsporozoite protein (CSP)^50,51^ led to phase I and II trials of the CSP-based RTS,S vaccine^52^. These successful trials led to the recently completed phase III trials of RTS,S^53^. This constituted a major advance in the vaccine field because they established RTS,S as the first successful vaccine in humans against a parasite. However, the relatively modest efficacy (30–50%) of RTS,S was not surprising in light of the known complexity of the human immune response to *P. falciparum* in endemic populations. Hence, work continues on the development of more effective vaccines. Understanding the human immune response to *P. falciparum* infection will be key to the development of vaccines with improved efficacy and safety.

### Treatment

Treatment with antimicrobials is designed to clear infection or reduce infectious load, decrease disease severity, and ideally to provide a degree of secondary prevention against the nervous system effects of the infection. For viral infections, with the exception of HSV and HIV, there is often no specific treatment. Even for HSV encephalitis, standard treatment (intravenous acyclovir) is unavailable in many parts of the world. Cost effectiveness and stakeholder analyses could be useful in influencing policymakers to increase availability of antiviral treatment. For HIV-associated opportunistic infections such as cryptococcal meningitis, development of therapies that do not rely on intravenous administration is a priority because capacity for intravenous medication is limited in many LMICs, particularly in rural areas. There is also a need for improved access to new assays that detect antiretroviral therapy resistance (for example, the oligonucleotide ligase assay), as these can guide cART treatment. For many parasitic infections, antiparasitic medications are available, but their efficacy in reducing the neurological or cognitive sequelae remains uncertain. For this reason, as noted in the pathogenesis section, development of adjunctive therapies that target prevention or reduction of nervous-system injury is an important research priority.

Development of low-cost, low-toxicity antimicrobials that work against drug-resistant pathogens is a research priority for several infections, including tuberculous meningitis and neonatal sepsis caused by multiresistant Gram-negative infections. Qualitative studies to better understand medical non-compliance, and to develop innovative solutions to reduce non-compliance through newer technologies, such as mobile devices that support medical and public-health practice, are also needed.

Finally, there is a need for multicentre clinical research trials with sufficient sample sizes to provide definitive answers on the efficacy of specific interventions. For example, where smaller trials had failed, the Cryptococcal Optimal Anti-Retroviral Timing (COAT) trial conducted in Uganda and South Africa successfully determined that deferred initiation of anti-retroviral therapy in individuals with HIV until 5 weeks after treatment of cryptococcal meningitis improved survival^54^. This study finding is likely to change international guidelines.

### Physical, occupational and cognitive rehabilitation

Whereas physical, occupational and cognitive rehabilitation for individuals with sequelae of CNS infections are routine in developed countries, such interventions are limited in LMICs owing to a lack of trained personnel and prohibitive costs. Thus, research on how to build capacity for rehabilitation and how to support it in the context of LMICs is required. Rehabilitation for cognitive impairment can be successfully implemented in the community using locally available resources or in a tertiary institution using advanced methods. In the community, home stimulation, parenting education and support, and provision of financial support or nutritional support for children enrolled in early child development centres have shown some benefit in improving children's cognition^55,56^. Interventions that target both the carer and the child are more effective than those that include either one^56^. In tertiary centres, computer-based cognitive training programmes have proven effective in improving cognition in African children surviving CNS infections^57,58^. These cognitive training programmes can target specific disabilities; however, they are in their early stages and more research is required to determine the most cost-effective implementable and sustainable programmes for LMICs.

### Operations and implementation research

Operationalization and implementation of known effective interventions is another research priority area. Vaccines for *S. pneumoniae* and *N. meningiditis* are highly effective and have been implemented in some LMICs, but these vaccine-preventable infections continue to affect more than 1 million people each year. Thus, in addition to increased investment in the basic science of vaccines, a major research priority is the assessment of methods to support and implement widespread vaccination in LMICs.

Another example is implementation research related to effective prenatal, perinatal and neonatal care, which would decrease neonatal sepsis. Assessment of effective methods for non-physician health workers to provide medical and preventive care to mothers and newborns in LMICs are needed^59^. The recent increase in neonatal infection with multi-drug-resistant Gram-negative organisms in LMICs^24,25^ makes prevention of neonatal sepsis an even greater research priority. Rapid diagnostics and treatment interventions that are successful in field trials also require implementation research for successful wide-scale adoption and appropriate use.

### Capacity building

Capacity building within LMICs is key to the successful reduction or elimination of nervous system complications of infection. The Review on research capacity building in this collection addresses this topic in depth (see page S207). Research priorities specifically in the area of infection-related nervous system morbidity include an increase in the number of clinicians and researchers in infectious disease, neuroscience, neurology and mental health^60^, and the dedication of a portion of LMICs health budgets to infectious disease and mental and neurological health^61^ . Research training grants and collaborative research between partners in LMICs and high-income countries specifically in the area of infection-related neurocognitive impairment can also help to build human resource capacity. Physical infrastructure is another priority; without space for laboratories, diagnostic equipment or research clinics, surveillance cannot be performed and information to guide interventions to reduce the burden of these infections cannot be generated. With improved human resource capacity and infrastructure, the development of effective screening instruments, prevention and treatment, and increased government support to address these infections are more likely to be achieved. Box 2 provides examples of capacity building in Peru and Uganda in the area of infections that affect the nervous system. This was enabled by Fogarty International Center grants for collaborative US–LMICs partnerships in these countries.

## CONCLUSIONS

In the past two decades, an increasing body of evidence implicates infection as a cause of substantial nervous system morbidity in high-income countries and LMICs. The burden is particularly high in LMICs, where infections such as HIV (and associated opportunistic infections), HSV, dengue, bacterial and tuberculous meningitis, malaria, neurocysticercosis, STH, schistosomiasis and other infections affect billions of people annually, and cause substantial neurological morbidity in those individuals. The involvement of the nervous system in infections is often first recognized in LMICs where the prevalence of these infections is higher and where new infections often emerge. Research conducted in these countries can contribute to prevention and cure before these infections become globalized. Research is needed in multiple areas to determine the true burden of disease and to develop point-of-care diagnostic assays for diagnosing infection, vaccines and other interventions for preventing infections; to improve our understanding of the pathogenesis of nervous system disease in these infections; to develop better tools for the assessment of neurological, cognitive and mental health impairment; to develop more effective treatments and preventions for nervous system sequelae, to improve the implementation of successful interventions, and to improve rehabilitation for those with long-term neurocognitive or mental health disabilities. Good research studies in these areas, accompanied by equally strong efforts to implement promising technologies and therapies, could substantially decrease the morbidity and mortality of infections affecting the nervous system in LMICs.

## Supplementary Material

Supplementary material 1

Supplementary material 2

## Figures and Tables

**Table 1:** Neurocognitive and mental health consequences of major infectious diseases that affect the nervous system.

Infectious disease	Regions affected	Estimated prevalence or annual incidence of infection	Health consequences		
			Neurological	Cognitive	Mental health
**VIRAL**					
Arboviruses					
Dengue virus	Global, most common in South Asia, Africa and Latin America	390 million (95% CI, 284-528 million)	• Meningitis, meningoencephalitis, encephalitis, seizures, Guillain-Barré syndrome, neuralgic amyotrophy, hypokalaemic paralysis, and dengue myositis • In one cohort, dengue had neurological manifestations in 9.3% of children and adults • There is limited information about long-term sequelae in dengue, but there is evidence of significant long-term neurological complications	• Not studied	Case reports of mania and depression
Chikungunya virus	Global, most common in South Asia, Africa and Latin America	33,000-93,000	• Encephalitis, febrile seizures, meningismus, myelopathy or myeloneuropathy	• Not studied	Not studied
Japanese encephalitis	Southeast Asia	35,000-50,000	• CNS complications during the acute illness include delirium, seizures, axial rigidity, extrapyramidal signs, cranial nerve palsies, ataxia, paraplegia and segmental sensory disturbances	• Among survivors, 30-50% have significant neurological, cognitive or psychiatric sequelae	Among survivors, 30-50% have significant neurological, cognitive, or psychiatric sequelae
Rhabdoviruses					
Rabies	Global, greatest in sub-Saharan Africa, Southeast Asia and Latin America	60,000 (probably an underestimate)	• Severe encephalitis, which is almost 100% fatal	• Fatal	Fatal
Herpesviruses					
HSV encephalitis	Global	Present in all countries where HSV testing has been performed, but no reliable global estimates	• If untreated, as in most LMICs, there is a high fatality rate for HSV-1 (around 70%), lower (around 15%) if treated. Long-term neurological complications occur in around 70% of adult survivors, including seizure disorder and hemiparesis. In one cohort, neurological sequelae occurred in 63% of infections in children, including seizures in 44% and developmental delays in 25%	• In one study of adult survivors, long-term cognitive sequelae included memory impairment (69%)	Personality or behavioural impairment in 45% of adult survivors
VZV	Global	No reliable global estimates	• CNS: stroke, meningoencephalitis, myelitis • PNS (more common): herpes zoster with chronic pain	• Very limited studies with conflicting results	Major depression
Congenital cytomegalovirus	Global	0.6-0.7% of live births in high-income countries and 1-5% of live births in LMICs	• Most common non-hereditary cause of hearing loss in children in the United States • There are no reliable estimates for frequency of hearing loss due to the infection in most LMICs	• Symptomatic infection, seen in 10-15% of congenitally infected children, is associated with significant global developmental delay in around 50% of affected children	Behavioural problems
HIV-related					
HIV	Global, greatest burden in sub-Saharan Africa and Asia	Annual incidence estimate is 2.3 million (95% CI, 1.9-2.7 million) with 34 million people living with HIV/AIDS worldwide, of whom 23 million live in sub-Saharan Africa and 3.5 million live in Southeast Asia	• HIV associated opportunistic infections, aseptic meningitis, AIDS encephalopathy, Bell's palsy, progressive multifocal leukoencephalopathy, primary CNS lymphoma, stroke, transverse myelitis, HIV-associated peripheral neuropathy, inflammatory demyelinating polyneuropathy, immune reconstitution inflammatory syndrome and vacuolar myelopathy	• Asymptomatic neurocognitive impairment, mild neurocognitive disorder and HIV-associated dementia	Delirium, major depression, bipolar disorder (including AIDS mania), schizophrenia, substance abuse or dependence and post-traumatic stress disorder
Cryptococcal meningitis	Global, greatest burden in sub-Saharan Africa and Asia	Annual incidence estimate: 957,900 in 2009, approximately 624,700 deaths annually	• Headache, meningismus, intracranial hypertension, mental status changes, focal intracerebral granulomas (cryptococcomas), hydrocephalus (communicating and non-communicating), papilledema, sensorineural deafness, cranial nerve palsies, motor and sensory deficits, cerebellar dysfunction and seizures	• Mimicking of vascular dementia, and reversible dementia	Personality change, confusional psychosis and mania
Toxoplasma encephalitis	Global, greatest burden in sub-Saharan Africa and Asia	No reliable global estimates of incidence of toxoplasma encephalitis, but toxoplasma infection is present in 14% of the population in the United States, compared with 23-47% in some European, Latin American and African countries	• Headache, focal neurological deficit, seizures and altered mental status	• Dementia	Schizophrenia and behaviour disorders
**BACTERIAL**					
Neonatal sepsis and meningitis	Global	Annual incidence estimates for south Asia, sub-Saharan Africa and Latin America: neonatal sepsis, 1.7 million (uncertainty estimate, 1.1-2.4 million); neonatal meningitis, 200,000; 95% CI, 21,000-350,000)	• Little data for neonatal sepsis globally, especially among those more than 32 weeks gestation or more than 1,500g • 23% (95% CI, 19-26%) of neonatal meningitis survivors (or 18,000 children; 95% CI, 2,700-35,000) estimated to sustain moderate to severe neurodevelopmental impairment • In sepsis or meningitis, the primary neurological sequelae are cerebral palsy, impairment to vision, hearing and motor function, and seizure disorders	• Limited studies reporting cognitive impairment; developmental delay or learning difficulties are frequent in sepsis (30.0%; IQR, 26.4-44.4%) and meningitis (33.3%; IQR, 26.7-36.8%)	No data
Bacterial meningitis	Global	Annual incidence estimate: 1.2 million	• 22.8% (IQR, 12.1-29.2%) have at least 1 neurocognitive sequela at discharge, 19.9% (IQR, 12.1-35.2%) have at least 1 sequela post-discharge; 16.0% (IQR, 7.1-21.2%) have at least 1 major sequela at discharge, 12.8% (iQr, 7.1-21.1%) have at least 1 major sequela post discharge • Neurological sequelae include motor deficits, hearing loss and visual disturbances • Risk of major sequelae is higher in Africa (25.1%) and southeast Asia (21.6%) compared with Europe (9.4%)	• In children, cognitive impairment including low IQ, academic limitations, and impared executive function and in adults, cognitive impairment with slower cognitive speed seen	Behavioural changes and emotional disturbance including ADHD and learning difficulties
Tuberculous meningitis (also an opportunistic infection in HIV)	Global, most burden in sub-Saharan Africa and Asia	No reliable global incidence estimates; highest in countries with high prevalence of HIV infection	• Neurological sequelae in 53.9% of child survivors (95% CI, 42.6-64.9) • Gross and fine motor impairment in children • Motor deficits, optic atrophy, ophthalmoplegia, and hearing impairment in adults and older children	• Cognitive impairment in all areas tested, and poor scholastic progress	Emotional disturbance
Neurosyphilis	Global	No reliable global incidence estimates; most cases occur in HIV-positive individuals	• Meningitis, cerebrovascular infarction, and paresis, tabes dorsalis (ataxia, paraesthesia and bladder dysfunction)	• Impaired memory, disorientation and dementia	Dementia, depression, delirium, mania and psychosis
**PARASITIC**					
Neurocysticercosis	Global, greatest burden in pig-raising areas with poor sanitation	2010 prevalence estimate: 1.4 million (95% CI, 1.3-1.6 million) (epilepsy only)	• Among people with symptomatic neurocysticercosis diagnosed with brain imaging: seizures and epilepsy (78.8%; 95% CI, 65.1-89.7%), headaches (37.9%; 95% CI, 23.3-53.7%), focal deficits (16.0%; 95% CI, 9.7-23.6%) and symptoms associated with increased intracranial pressure (11.7%; 95% CI, 6.0-18.9%)	• Case reports of cognitive decline • Cognitive symptoms of neurocysticercosis with active cysts: affects naming, verbal fluency and non-verbal memory	Neurocysticercosis with active cysts: dementia (12.5%) and cognitive impairment, but not dementia (27.5%); psychosis
Malaria	Sub-Saharan Africa, Latin America, Asia and Oceania	Annual incidence estimate: 216 million	• Cerebral malaria: 5-28% of children have neurological deficits on discharge, including epilepsy, acute hemiparesis, hypertonia, cortical blindness and ataxia • By 6-month follow-up the percentage of children with deficits has decreased to 0-4.4%, primarily in the areas of gross motor and fine motor skills • Uncomplicated malaria: motor skills	• Cerebral malaria affects general cognition, attention, working memory, visual spatial skills, somatosensory discrimination, speech and language, and receptive and expressive language • Thirteen IQ point difference from non-affected children 1 year after episode, and around 26% of children have impairment 2 years after • Severe malaria with neurological involvement affects executive function • Severe malarial anaemia affects overall cognition estimated to lead to the equivalent of an 11 IQ point difference from non-affected community children • Malaria with multiple seizures leads to speech and language problems • Malaria with impaired consciousness leads to attention/language problems • Uncomplicated malaria leads to language problems • Asymptomatic malaria leads to problems with fine motor coordination, attention and abstract reasoning	Cerebral malaria: internalizing and externalizing problems, ADHD, disruptive behaviour, psychosis and depression
STH infection	Global, greatest burden in sub-Saharan Africa and Southeast Asia	Estimated 2010 prevalence: hookworm infected 439 million (95% CI, 406-480), *Ascaris lumbricoides* infected 819 million (95% CI, 772-892) and *Trichuris trichiura* infected 465 million (95% CI, 430-508).	• Not described	• School-aged children: *T. trichiura* and *A. lumbricoides* affected learning and verbal memory in one study • In another, general STH infection reduced memory capacity, rate of processing and attention	Children under 5 years of age: social and emotional disturbances (combined with anaemia)
Schistosomiasis	Global, greatest in sub-Saharan Africa and Southeast Asia	Estimated 2010 prevalence: 252 million infected	• Acute schistosomal encephalopathy: headache, confusion, seizure, loss of consciousness, focal deficits, visual impairment and ataxia • Cerebral schistosomiasis: headaches, motor deficits, visual abnormalities, seizures, altered mental status, vertigo, sensory impairment, speech disturbances and ataxia • Spinal cord schistosomiasis: lower limb weakness, bladder dysfunction, lower limb paraesthesia, hypoaesthesia/anaesthesia, deep tendon reflex abnormalities, constipation and impotence in 80% of cases	• For *Schistosoma japonicum* infection in children (not neurological infection): verbal memory and verbal fluency affected	No data

ADHD, attention deficit disorder; CI, confidence interval; CNS, central nervous system; HSV, herpes simplex virus; IQR, interquartile range; LMICs, low- and middle-income countries; PNS, peripheral nervous system; STH, soil-transmitted helminths; VZV, varicella-zoster virus. Prevalence estimates are typically used (for example, STH infections and schistosomiasis) because accurate incidence numbers for these infections are difficult to obtain.

**Table 2:** Potential areas for intervention in infectious diseases that affect the nervous system.

Disease	Vaccine available	Control of zoonotic reservoirs	Control of vector populations	Treatment

**VIRAL**				
Dengue	New dengue vaccines being tested in large field trials	NA	Yes	None available
Chikungunya	No	NA	Yes	None available
Japanese encephalitis	Yes	No	Yes	None available
Rabies	Yes	Yes	NA	None available
HSV encephalitis	No	NA	NA	Yes
VZV	Yes	NA	NA	Yes
Congenital cytomegalovirus	No	NA	NA	Yes
HIV-related				
HIV	No	NA	NA	Yes
Cryptococcal meningitis	No	NA	NA	Yes
Toxoplasma encephalitis	No	Yes	NA	Yes
**BACTERIAL**				
Neonatal sepsis and meningitis	No	NA	NA	Yes
Bacterial meningitis	Yes, for *Haemophilus influenzae* type b, and pneumococcal (multiple serotypes) and meningococcal (A, C, Y and W135) meningitis	NA	NA	Yes
Tuberculous meningitis	Partial protection provided by BCG vaccination	Infrequent (cases due to *Mycobacterium bovid* and *Mycobacterium caprae,* both of which are present in cattle, reported)	NA	Yes
Neurosyphilis	No	NA	NA	Yes
**PARASITIC**				
Neurocysticercosis	No	Porcine vaccine trials underway, pig treatment available	NA	Yes
Malaria	RTS,S vaccine had efficacy in phase III studies and other vaccines are being developed	NA except for *Plasmodium knowlesi*	Yes	Yes
STH	No. Hookworm vaccine is in phase I trials, but is linked to adverse events	NA except for *Toxocara canis*	NA	Yes
Schistosomiasis	No, but phase I vaccine trials are ongoing	Bovine vaccine trials underway for *Schistosoma japonicum*	Yes	Yes

BCG, Bacillus Calmette-Guerin; HSV, herpes simplex virus; NA, not applicable; STH, soil-transmitted helminth; VZV, varicella-zoster virus.

**Table 3:** Global research for infections that affect the nervous system.

Priority area	Research needed
**Diagnosis**	• Rapid, accurate, low-cost, point-of-care diagnostic tests for infections that affect the nervous system • Clinical diagnostic algorithms for infections that affect the nervous system • Improved testing for detection of infection-related nervous-system disabilities
**Epidemiology**	• Accurate incidence and prevalence estimates of common infections that affect the nervous system • Accurate identification and frequency estimates of nervous-system manifestations and sequelae • Identification of potentially modifiable risk factors specific to infections that affect the nervous system
**Pathogenesis**	• Identification of host response pathways that lead to nervous-system deficits or to clinical immunity • Identification of pathogen factors that lead to nervous-system deficits or to clinical immunity • Assessment of risks and interactions of co-infections and co-morbidity
**Vaccine development**	• Develop safe and effective vaccines based on immunology, epidemiology and pathogenesis studies • Phase I and II trials • Phase III trials
**Treatment**	• Effective adjunctive treatment to prevent or decrease nervous-system deficits or disabilities • Low cost, low toxicity antimicrobials that work against drug-resistant pathogens • Multi-site, large clinical trials that provide definitive answers on interventions • Effective or improved primary treatment of infection
**Rehabilitation**	• Effective and feasible physical, occupational and cognitive rehabilitation programmes
**Operations and implementation**	• Optimal methods to implement or operationalize interventions with known efficacy

## BOX 1 | EMERGING RESEARCH AREAS

Diarrhoeal disease

Diarrhoea is a leading cause of mortality in children living in low- and middle-income countries (LMICs)^62^. Most of these deaths occur in Africa, Southeast Asia and in eastern Mediterranean countries^63^. In 2010, it is estimated that there were 1.731 billion episodes of diarrhoea worldwide of which 36 million progressed to severe diarrhoea and 700,000 episodes resulted in death^64^. Its occurrence in the first 2 years of life is associated with an 8 cm decrease in height and a 10 point drop in IQ by the time children are around 7 to 9 years old^65^. The mechanism by which diarrhoea affects cognition is not clear, but it could be through the effect of diarrhoea on stunting, which in turn predicts future cognition^66^. However, during diarrhoea-free periods in the first 2 years of life, children experience catch-up growth and may return to their original growth trajectories^67^. This highlights the importance of effective interventions for diarrhoea to sustain the child's developmental potential. The high frequency of diarrhoea episodes during this critical developmental stage and the large number of cases makes diarrhoea a major public health concern for child development.

The microbiome

Although it has been clearly demonstrated that pathogenic microbes can cause brain disorders, there is increasing evidence that the microbial population harboured in the human body, termed the human microbiome, can as a whole influence brain activity^68^. Recent clinical studies among healthy subjects suggest that treatment with a probiotic is associated with reduced symptoms of stress and depression^69^. There is also evidence of associations between the microbiome and neurological diseases, such as multiple sclerosis and autism spectrum disorder (ASD)^70^. In a recent study using a mouse model of ASD, treatment with probiotics alleviated some behavioural symptoms of the disorder^71^. The composition of the human microbiome shows marked differences between countries^72^ and comparative research conducted in high-income countries and LMICs could lead to a better understanding of the part played by the human microbiome in brain disorders, and possible treatment of these disorders with factors that favourably alter the microbiome.

## BOX 2 | CAPACITY BUILDING IN UGANDA AND PERU

Uganda

The Severe Malaria Research Centre in Uganda is an example of how collaborations between local and foreign scientists, with support from the Fogarty International Center, has built a hub for research excellence. Through a National Institutes of Health (NIH) R21 exploratory research grant in 2004, local scientists developed research capacity by involvement in research studies, and grant and manuscript writing. This has since led to further NIH grants (four R01 grants, a U01 grant, a D43 grant and two R34 grants) as well as multiple grants from other agencies. Ugandan and US faculty are principal investigators on these grants. A book on neuropsychology of African children and more than 30 research papers have been published from these projects so far. Ugandan scientists and physicians have obtained faculty positions in Makerere University, Kampala. The infrastructure that has been built includes high-speed Internet connectivity for research offices and faculty members, a laboratory, a data room and a grants management office. With the infrastructure in place, this centre is now providing training for many Ugandan and US students and researchers at all levels of training, from undergraduates to post-doctoral fellows and faculty.

Peru

Through the Fogarty International Center NIH R21 and R01 grants in Peru, a network of neurologists who are engaged in brain-disorder research has been developed throughout the country. Trainee alumni of this network now serve as collaborators on emerging research and training activities in both infectious and chronic diseases of the nervous system (such as cerebrovascular diseases). The 2 sites in Lima have been scaled up to 12 hospitals and 2 universities in 3 Peruvian regions. Capacity building of individuals was provided through workshops, hybrid virtual/in-person certificate courses, as well as medium- and long-term training in Seattle and Peru. An initial mentor-training workshop developed into a growing network of mentors, three of whom have been awarded Clayton–Dedonder Mentorship Fellowships by the Fogarty International Center and have started institutionalization of mentor training programmes at three institutions in Lima. Those who received the R21 and R01 grants in the past are now experienced researchers who are leading the development of research in new areas, such as neurogenetics research and the development of a cerebrovascular diseases research training programme. Research supported by these awards resulted in 26 peer-reviewed publications and book chapters. Programme alumni are becoming leaders in brain research and are mentoring the newest wave of young neurologists and neuroscientists.
