# Supplementary material for: Global research priorities for infections that affect the nervous system
Source: Nature. Author manuscript; Available in PMC 2016 May 19. (PMC4697933; doi:10.1038/nature16033)
Supplement: Supplementary material 1 [file NIHMS743819-supplement-Supplementary_material_1.pdf]

Supplemental Table 1. Neurocognitive and mental health consequences of major infectious diseases that affect the nervous system, with references

			Areas Affected		
Infectious disease	Regions affected	Estimated prevalence or annual incidence of infection[a]	Neurologic	Cognitive	Mental health
**VIRAL**					
**Arboviruses**					
Dengue and Chikungunya virus infection	Global, most common in South Asia, Africa and Latin America	Dengue: 390 million (95% CI, 284-528)[1]; Chikungunya 33,000-93,000[2];	Dengue can present with meningitis, meningoencephalitis, encephalitis, seizures, Guillain-Barre syndrome, neuralgic amyotrophy, hypokalemic paralysis, and dengue myositis [3,4]<br>Dengue had neurologic manifestations in 9.3% of children and adults in one cohort[3,4]. Chikungunya can present as encephalitis, febrile seizures, meningismus, myelopathy or myeloneuropathy [5,6] Limited information about long-term sequelae in dengue and Chikungunya, but evidence of significant long-term neurologic complications in both illnesses [2].	Not studied	Dengue: Case reports of mania[7] and depression [8]
Japanese encephalitis	SEA	35,000 – 50,000 [2]	CNS complications during the acute illness include delirium, seizures, axial rigidity, extrapyramidal signs, cranial nerve palsies, ataxia, paraplegia and segmental sensory disturbances[9,10]	The mortality rate is 20%–30% and among survivors, 30%–50% have significant neurologic, cognitive, or psychiatric sequelae.[9]	The mortality rate is 20%–30% and among survivors, 30%–50% have significant neurologic, cognitive, or psychiatric sequelae[9]
**Rhadoviruses**					
Rabies	Global, greatest in SSA, SEA, Latin America	60,000 (probably underestimate [11])	Severe encephalitis; ~100% fatal	Fatal	Fatal
**Herpesviruses**					
HSV	Global	Present in all	If untreated, as in	6-month mortality	Memory

encephalitis		countries where HSV testing has been performed, but no reliable global estimates	most LMIC, high fatality rate for HSV-1 (~70%), lower (~15%) if treated[12]. Long term neurologic complications occur in ~70% of adult survivors, including memory impairment, seizure disorder, personality change and hemiparesis[13]. Neurologic sequelae occurred in 63% of pediatric infections, including seizures in 44% and developmental delays in 25%[14].	is about 19%. In one study of adult survivors, long term neurologic sequelae included memory impairment (69%), personality/behavioral impairment (45%), epilepsy (24%) and unilateral or bilateral anosmia (65%)[13]	impairment, personality/behavioral impairment
Varicella zoster virus infection	Global	No reliable global estimates	CNS: stroke, meningoencephalitis, myelitis. Peripheral NS (more common): herpes zoster, with chronic pain[15]	Limited information	Major depression[16]
Congenital cytomegalovirus infection	Global	0.6% to 0.7% of live births in high income countries and 1% to 5% of live births in LMIC[17].	Most common nonhereditary cause of hearing loss in children in the US[18]. No reliable estimates for frequency of hearing loss due to CMV in most LMIC	Symptomatic infection, seen in 10-15% of congenitally infected children, is associated with significant global developmental delay in ~50% of affected children [19]	Behavioral problems [20]
**HIV-RELATED**					
HIV	Global, greatest burden in SSA and Asia	34 million people are living with HIV/AIDS worldwide, of whom 23 million live in sub-Saharan Africa and 3.5 million live in Southeast Asia[21]. Annual incidence estimate: 2.3 million (1.9-2.7 million) [22]	HIV associated opportunistic infections, aseptic meningitis, AIDS encephalopathy, Bell palsy, progressive multifocal leukoencephalopathy (PML)[23], primary CNS lymphoma, stroke[24], transverse myelitis, HIV associated peripheral neuropathy, inflammatory demyelinating polyneuropathy[25], immune reconstitution inflammatory syndrome, vacuolar myelopathy[26]	Asymptomatic neurocognitive impairment (ANI), mild neurocognitive disorder (MND), and HIV-associated dementia (HAD)[27,28]	Delirium, minor cognitive-motor disorder (MCMD), major depression[29], bipolar disorder (including AIDS mania), schizophrenia, substance abuse or dependence, posttraumatic stress disorder (PTSD)[30]

Cryptococcal meningitis	Global, greatest burden in SSA and Asia	Annual incidence estimate: 957,900 in 2009[31], approximately 624,700 deaths annually[32]	Headache, meningismus, intracranial hypertension, mental status changes, focal intracerebral granulomas (cryptococcomas), hydrocephalus (both communicating and noncommunicating), papilledema, sensorineural deafness, cranial nerve palsies, motor and sensory deficits, cerebellar dysfunction, and seizures[31,33]	Vascular dementia mimicking,[34] reversible dementia[35,36]	Personality change, confusional psychosis, and mania[37]
Toxoplasma encephalitis	Global, greatest burden in SSA and Asia	Toxoplasma infection present in 14% of the population in the United States, versus 23-47% in certain European, Latin American, and African countries (FURTADO). No reliable global estimates of incidence of toxoplasma encephalitis.	Headache, focal neurologic deficit, cognitive dysfunction, seizures, and altered mental status[38]	Dementia[39]	Schizophrenia[40], behavior disorders[41]
**BACTERIAL**					
Neonatal sepsis and meningitis	Global	Annual incidence estimates for South Asia, sub-Saharan Africa and Latin America: Neonatal sepsis, 1.7 million (uncertainty estimate, 1.1-2.4 million); neonatal meningitis, 200,000 (21,000-350,000) [42]	Little data for neonatal sepsis globally, especially among children >32 weeks gestation or >1,500 g; 23% (95% CI, 19-26%) of neonatal meningitis survivors (or 18,000 (95% CI 2,700-35,000) children) estimated to sustain moderate to severe NDI[42]. In sepsis or meningitis, primary neurologic sequelae are cerebral palsy, impairment in vision, hearing, motor function, and seizure disorders[43]	In limited studies, cognitive impairment, developmental delay or learning difficulties frequent in sepsis (30.0%, IQR 26.4-44.4) and meningitis (33.3%, IQR 26.7-36.8)[43]	No data
Bacterial meningitis	Global	Annual incidence estimate: 1.2 million [44]	22.8% (IQR, 12.1-29.2%) have ≥1 neurocognitive sequela at discharge,	Cognitive impairment including low IQ, academic	Behavioral changes, emotional disturbance [46]

			19.9% (IQR 12.1-35.2%) have ≥1 sequela post-discharge; 16.0% (7.1-21.2%) have ≥1 major sequela at discharge, 12.8% (7.1-21.1%) have ≥1 major sequela post discharge. Neurologic sequelae include motor deficits, hearing loss, visual disturbances. Risk of major sequelae higher in Africa (25.1%) and southeast Asia (21.6%) compared to Europe (9.4%) [45]	limitations, ADHD and mental retardation[46]; impaired intellectual, academic and executive ability[47]; in adults, cognitive impairment with slower cognitive speed seen[48,49]	
Tuberculous meningitis (also an opportunistic infection in HIV)	Global, greatest burden in SSA and Asia	No reliable global incidence estimates; highest in countries with high prevalence of HIV infection	Neurological sequelae in 53.9% of child survivors (95% CI 42.6-64.9)[50]. Gross and fine motor impairment in children[51]. Motor deficits, optic atrophy, ophthalmoplegia, and hearing impairment in adults and older children[52].	Cognitive impairment in all areas tested, poor scholastic progress[53]	Emotional disturbance [53]
Neurosyphilis	Global	No reliable global incidence estimates; most cases occur in HIV-positive individuals	Meningitis, cerebrovascular infarction, paresis, tabes dorsalis (ataxia, paresthesia, bladder dysfunction) [54]	Impaired memory, disorientation, dementia[54]	Dementia, depression, delirium, mania, psychosis[54]
**PARASITIC**					
Neurocysticer cosis	Global, greatest burden in pig-raising areas with poor sanitation	2010 prevalence estimate: 1.4 million (95%CI: 1.3-1.6 million) (epilepsy only) [55]	Among people with symptomatic NCC diagnosed with brain imaging: seizures and epilepsy (78.8%, 95%CI: 65.1%–89.7%), headaches (37.9%, 95%CI: 23.3%–53.7%), focal deficits (16.0%, 95%CI: 9.7%–23.6%) and symptoms associated with increased intracranial pressure (11.7%, 95%CI: 6.0%–18.9%) (see systematic review in[56])	Case reports: Cognitive decline[57]; cognitive symptoms [58], NCC with active cysts: naming, verbal fluency, nonverbal memory[56]	NCC with active cysts: Dementia (12.5%) and cognitive impairment not dementia (27.5%)[59]; psychosis [56]
Malaria	SSA, Latin America, Asia, Oceania	Annual incidence estimate: 216 million [60]	Cerebral malaria: 5 to 28% of children have neurologic deficits on discharge[61,62]; this includes epilepsy [63,64];	Cerebral malaria: general cognition, attention, working memory, visual spatial skills,	Cerebral malaria: internalizing and externalizing problems, ADHD, disruptive

			acute - hemiparesis, hypertonia, cortical blindness, ataxia [64-69] by 6 month follow-up, % of children with deficits has decreased to 0% to 4.4%[61,70] ,primarily in the areas of gross motor and fine motor skills [69] Uncomplicated malaria: motor skills[71]	somatosensory discrimination, speech and language, receptive and expressive language [72-80] Estimated 13 IQ point difference from community children 1 year after episode[81], and ~26% of children have impairment 2 years after episode[77] Severe malaria with neurological involvement: Executive function[82] Severe malarial anemia: overall cognition [80]. Estimated to lead to equivalent of 11 IQ point difference from community children[81]. Malaria with multiple seizures; speech and language, [75,76] Malaria with impaired consciousness: attention and language [83] Uncomplicated malaria: language [84-87] Asymptomatic malaria: fine motor coordination, attention, abstract reasoning [88,89]	behavior, psychosis and depression [63,64,67,90-92]
Soil-transmitted helminth infection	Global, greatest burden in SSA and SEA	2010 prevalence estimates : Hookworm: 439 million (95%CI: 406-480) *A lumbricoides*: 819 million (772-892) *T trichuria* : 465 million (430-508)[93]	Not described	Infant and pre-school children: social and emotional disturbances (in combination with anemia) [94] School-aged children for *T. trichuria* and *A. lumbricoides*: Learning and verbal memory[95]; STH: reduced memory	No data

				capacity, rate of processing and attention[94]	
Schistosomiasis	Global, greatest in SSA and SEA	2010 prevalence estimate : 252 million[96]	Acute schistosomal encephalopathy (ASE): headache, confusion, seizure, loss of consciousness, focal deficits, visual impairment, ataxia[97,98]; Cerebral schistosomiasis (based on 21 patients from 2 case series): Headaches (100%), motor deficits (71%), visual abnormalities (62%), seizures (57%), altered mental status (57%), vertigo (43%), sensory impairment (38%), speech disturbances (29%), ataxia (24%)[98]. Spinal cord schistosomiasis: in 80% of cases: lower limb weakness, bladder dysfunction, lower limb paraesthesia, hypoaesthesia or anastesia, deep tendon reflex abnormalities, constipation, impotence [98]	For *S. japonicum* infection in children (not neurological infection): Verbal memory and verbal fluency[95,99]	No data

Abbreviations: SSA: Sub-Saharan Africa; SEA: Southeast Asia

[a] Prevalence estimates used for infections that are typically chronic (e.g., soil-transmitted helminth infections, schistosomiasis), as accurate incidence numbers for these infections are difficult to obtain.

REFERENCES

1 Bhatt, S. *et al.* The global distribution and burden of dengue. *Nature* **496**, 504-507, doi:10.1038/nature12060 (2013).

2 Labeaud, A. D., Bashir, F. & King, C. H. Measuring the burden of arboviral diseases: the spectrum of morbidity and mortality from four prevalent infections. *Popul Health Metr* **9**, 1, doi:10.1186/1478-7954-9-1 (2011).

3 Sahu, R. *et al.* Neurologic complications in dengue virus infection: A prospective cohort study. *Neurology*, doi:10.1212/wnl.0000000000000935 (2014).

4 Verma, R., Sahu, R. & Holla, V. Neurological manifestations of dengue infection: A review. *J Neurol Sci*, doi:10.1016/j.jns.2014.08.044 (2014).

5 Robin, S. *et al.* Neurologic manifestations of pediatric chikungunya infection. *J Child Neurol* **23**, 1028-1035, doi:10.1177/0883073808314151 (2008).

6 Tandale, B. V. *et al.* Systemic involvements and fatalities during Chikungunya epidemic in India, 2006. *J Clin Virol* **46**, 145-149, doi:10.1016/j.jcv.2009.06.027 (2009).

7 Srivastava, S., Bhatia, M. S. & Jhanjee, A. Organic mania in dengue. *J Clin Diagn Res* **7**, 566-567, doi:10.7860/jcdr/2013/4891.2827 (2013).

8 Hashmi, A. M. *et al.* Anxiety and depression symptoms in patients with dengue fever and their correlation with symptom severity. *Int J Psychiatry Med* **44**, 199-210 (2012).

9 Richter, R. W. & Shimojyo, S. Neurologic sequelae of Japanese B encephalitis. *Neurology* **11**, 553-559 (1961).

10 Lowry, P. W. *et al.* Japanese encephalitis among hospitalized pediatric and adult patients with acute encephalitis syndrome in Hanoi, Vietnam 1995. *Am J Trop Med Hyg* **58**, 324-329 (1998).

11 Fooks, A. R. *et al.* Current status of rabies and prospects for elimination. *Lancet* **384**, 1389-1399, doi:10.1016/S0140-6736(13)62707-5 (2014).

12 Barza, M. & Pauker, S. G. The decision to biopsy, treat, or wait in suspected herpes encephalitis. *Ann Intern Med* **92**, 641-649 (1980).

13 McGrath, N., Anderson, N. E., Croxson, M. C. & Powell, K. F. Herpes simplex encephalitis treated with acyclovir: diagnosis and long term outcome. *J Neurol Neurosurg Psychiatry* **63**, 321-326 (1997).

14 Elbers, J. M. *et al.* A 12-year prospective study of childhood herpes simplex encephalitis: is there a broader spectrum of disease? *Pediatrics* **119**, e399-407, doi:10.1542/peds.2006-1494 (2007).

15 Kleinschmidt-DeMasters, B. K. & Gilden, D. H. Varicella-Zoster virus infections of the nervous system: clinical and pathologic correlates. *Archives of pathology & laboratory medicine* **125**, 770-780, doi:10.1043/0003-9985(2001)125<0770:VZVIOT>2.0.CO;2 (2001).

16 Chen, M. H. *et al.* Risk of depressive disorder among patients with herpes zoster: a nationwide population-based prospective study. *Psychosomatic medicine* **76**, 285-291, doi:10.1097/PSY.0000000000000051 (2014).

17 Manicklal, S., Emery, V. C., Lazzarotto, T., Boppana, S. B. & Gupta, R. K. The "silent" global burden of congenital cytomegalovirus. *Clin Microbiol Rev* **26**, 86-102, doi:10.1128/cmr.00062-12 (2013).

18 Swanson, E. C. & Schleiss, M. R. Congenital cytomegalovirus infection: new prospects for prevention and therapy. *Pediatr Clin North Am* **60**, 335-349, doi:10.1016/j.pcl.2012.12.008 (2013).

19 Boppana, S. B., Ross, S. A. & Fowler, K. B. Congenital cytomegalovirus infection: clinical outcome. *Clinical infectious diseases : an official publication of the Infectious Diseases Society of America* **57 Suppl 4**, S178-181, doi:10.1093/cid/cit629 (2013).

20 Saigal, S., Lunyk, O., Larke, R. P. & Chernesky, M. A. The outcome in children with congenital cytomegalovirus infection. A longitudinal follow-up study. *Am J Dis Child* **136**, 896-901 (1982).

21 *Panel on Opportunistic Infections in HIV-Infected Adults and Adolescents. Guidelines for the prevention and treatment of opportunistic infections in HIV-infected adults and adolescents: recommendations from the Centers for Disease Control and Prevention, the National Institutes of Health, and the HIV Medicine Association of the Infectious Diseases Society of America*, <http://aidsinfo.nih.gov/contentfiles/adult_oi.pdf> (

22 *Number of people (all ages) living with HIV*, <http://www.who.int/gho/hiv/epidemic_status/cases_all/en/> (2014).

23 Sahraian, M. A., Radue, E. W., Eshaghi, A., Besliu, S. & Minagar, A. Progressive multifocal leukoencephalopathy: a review of the neuroimaging features and differential diagnosis. *Eur J Neurol* **19**, 1060-1069, doi:10.1111/j.1468-1331.2011.03597.x (2012).

24 Benjamin, L. A. *et al.* HIV infection and stroke: current perspectives and future directions. *Lancet Neurol* **11**, 878-890, doi:10.1016/S1474-4422(12)70205-3 (2012).

25 Centner, C. M., Bateman, K. J. & Heckmann, J. M. Manifestations of HIV infection in the peripheral nervous system. *The Lancet Neurology* **12**, 295-309, doi:http://dx.doi.org/10.1016/S1474-4422(13)70002-4 (2013).

26 Singer, E. J., Valdes-Sueiras, M., Commins, D. & Levine, A. Neurologic presentations of AIDS. *Neurologic clinics* **28**, 253-275, doi:10.1016/j.ncl.2009.09.018 (2010).

27 Antinori, A. *et al.* Updated research nosology for HIV-associated neurocognitive disorders. *Neurology* **69**, 1789-1799, doi:10.1212/01.WNL.0000287431.88658.8b (2007).

28 Gisslen, M., Price, R. W. & Nilsson, S. The definition of HIV-associated neurocognitive disorders: are we overestimating the real prevalence? *BMC Infect Dis* **11**, 356, doi:10.1186/1471-2334-11-356 (2011).

29 Berger-Greenstein, J. A. *et al.* Major depression in patients with HIV/AIDS and substance abuse. *AIDS Patient Care STDS* **21**, 942-955, doi:10.1089/apc.2006.0153 (2007).

30 Johnson, J. G., Williams, J. B., Rabkin, J. G., Goetz, R. R. & Remien, R. H. Axis I psychiatric symptoms associated with HIV infection and personality disorder. *Am J Psychiatry* **152**, 551-554 (1995).

31 Makadzange, A. T. & McHugh, G. New approaches to the diagnosis and treatment of cryptococcal meningitis. *Semin Neurol* **34**, 47-60, doi:10.1055/s-0034-1372342 (2014).

32 McKenney, J. *et al.* Prevalence and correlates of cryptococcal antigen positivity among AIDS patients--United States, 1986-2012. *MMWR Morb Mortal Wkly Rep* **63**, 585-587 (2014).

33 Jarvis, J. N. & Harrison, T. S. HIV-associated cryptococcal meningitis. *Aids* **21**, 2119-2129, doi:10.1097/QAD.0b013e3282a4a64d (2007).

34 Aharon-Peretz, J. *et al.* Cryptococcal meningitis mimicking vascular dementia. *Neurology* **62**, 2135 (2004).

35 Ala, T. A., Doss, R. C. & Sullivan, C. J. Reversible dementia: a case of cryptococcal meningitis masquerading as Alzheimer's disease. *Journal of Alzheimer's disease : JAD* **6**, 503-508 (2004).

36 Hoffmann, M., Muniz, J., Carroll, E. & De Villasante, J. Cryptococcal meningitis misdiagnosed as Alzheimer's disease: complete neurological and cognitive recovery with treatment. *J Alzheimers Dis* **16**, 517-520, doi:10.3233/JAD-2009-0985 (2009).

37 Sa'adah, M. A., Araj, G. F., Diab, S. M. & Nazzal, M. Cryptococcal meningitis and confusional psychosis. A case report and literature review. *Trop Geogr Med* **47**, 224-226 (1995).

38 Porter, S. B. & Sande, M. A. Toxoplasmosis of the central nervous system in the acquired immunodeficiency syndrome. *N Engl J Med* **327**, 1643-1648, doi:10.1056/nejm199212033272306 (1992).

39 Habek, M., Ozretic, D., Zarkovic, K., Djakovic, V. & Mubrin, Z. Unusual cause of dementia in an immunocompetent host: toxoplasmic encephalitis. *Neurol Sci* **30**, 45-49, doi:10.1007/s10072-008-0007-5 (2009).

40 Torrey, E. F. & Yolken, R. H. Toxoplasma gondii and schizophrenia. *Emerg Infect Dis* **9**, 1375-1380, doi:10.3201/eid0911.030143 (2003).

41 Fekadu, A., Shibre, T. & Cleare, A. J. Toxoplasmosis as a cause for behaviour disorders--overview of evidence and mechanisms. *Folia Parasitol (Praha)* **57**, 105-113 (2010).

42 Seale, A. C. *et al.* Neonatal severe bacterial infection impairment estimates in South Asia, sub-Saharan Africa, and Latin America for 2010. *Pediatr Res* **74 Suppl 1**, 73-85, doi:10.1038/pr.2013.207 (2013).

43 Mwaniki, M. K., Atieno, M., Lawn, J. E. & Newton, C. R. Long-term neurodevelopmental outcomes after intrauterine and neonatal insults: a systematic review. *Lancet* **379**, 445-452, doi:10.1016/S0140-6736(11)61577-8 (2012).

44 van de Beek, D. Progress and challenges in bacterial meningitis. *Lancet* **380**, 1623-1624, doi:10.1016/S0140-6736(12)61808-X (2012).

45 Edmond, K. *et al.* Global and regional risk of disabling sequelae from bacterial meningitis: a systematic review and meta-analysis. *Lancet Infect Dis* **10**, 317-328, doi:10.1016/s1473-3099(10)70048-7 (2010).

46 Chandran, A., Herbert, H., Misurski, D. & Santosham, M. Long-term sequelae of childhood bacterial meningitis: an underappreciated problem. *Pediatr Infect Dis J* **30**, 3-6, doi:10.1097/INF.0b013e3181ef25f7 (2011).

47 Anderson, V., Anderson, P., Grimwood, K. & Nolan, T. Cognitive and executive function 12 years after childhood bacterial meningitis: effect of acute neurologic complications and age of onset. *J Pediatr Psychol* **29**, 67-81 (2004).

48 Hoogman, M., van de Beek, D., Weisfelt, M., de Gans, J. & Schmand, B. Cognitive outcome in adults after bacterial meningitis. *J Neurol Neurosurg Psychiatry* **78**, 1092-1096, doi:10.1136/jnnp.2006.110023 (2007).

49 Merkelbach, S., Sittinger, H., Schweizer, I. & Muller, M. Cognitive outcome after bacterial meningitis. *Acta Neurol Scand* **102**, 118-123 (2000).

50 Chiang, S. S. *et al.* Treatment outcomes of childhood tuberculous meningitis: a systematic review and meta-analysis. *Lancet Infect Dis* **14**, 947-957, doi:10.1016/s1473-3099(14)70852-7 (2014).

51 Schoeman, C. J., Herbst, I. & Nienkemper, D. C. The effect of tuberculous meningitis on the cognitive and motor development of children. *S Afr Med J* **87**, 70-72 (1997).

52 Kalita, J., Misra, U. K. & Ranjan, P. Predictors of long-term neurological sequelae of tuberculous meningitis: a multivariate analysis. *Eur J Neurol* **14**, 33-37, doi:10.1111/j.1468-1331.2006.01534.x (2007).

53 Schoeman, J. *et al.* Long-term follow up of childhood tuberculous meningitis. *Dev Med Child Neurol* **44**, 522-526 (2002).

54 Ghanem, K. G. REVIEW: Neurosyphilis: A historical perspective and review. *CNS Neurosci Ther* **16**, e157-168, doi:10.1111/j.1755-5949.2010.00183.x (2010).

55 Murray, C. J. *et al.* Disability-adjusted life years (DALYs) for 291 diseases and injuries in 21 regions, 1990-2010: a systematic analysis for the Global Burden of Disease Study 2010. *Lancet* **380**, 2197-2223, doi:10.1016/S0140-6736(12)61689-4 (2012).

56 Carabin, H. *et al.* Clinical manifestations associated with neurocysticercosis: a systematic review. *PLoS neglected tropical diseases* **5**, e1152, doi:10.1371/journal.pntd.0001152 (2011).

57 Shandera, W. X. & Kass, J. S. Neurocysticercosis: current knowledge and advances. *Curr Neurol Neurosci Rep* **6**, 453-459 (2006).

58 Shah, R. & Chakrabarti, S. Neuropsychiatric manifestations and treatment of disseminated neurocysticercosis: a compilation of three cases. *Asian J Psychiatr* **6**, 344-346, doi:10.1016/j.ajp.2013.01.013 (2013).

59 Rodrigues, C. L. *et al.* Spectrum of cognitive impairment in neurocysticercosis: differences according to disease phase. *Neurology* **78**, 861-866, doi:10.1212/WNL.0b013e31824c46d1 (2012).

60 White, N. J. *et al.* Malaria. *Lancet* **383**, 723-735, doi:10.1016/S0140-6736(13)60024-0 (2014).

61 Boivin, M. J. *et al.* Cognitive impairment after cerebral malaria in children: a prospective study. *Pediatrics* **119**, e360-366, doi:10.1542/peds.2006-2027 (2007).

62 Idro, R., Karamagi, C. & Tumwine, J. Immediate outcome and prognostic factors for cerebral malaria among children admitted to Mulago Hospital, Uganda. *Ann Trop Paediatr* **24**, 17-24, doi:10.1179/027249304225013240 (2004).

63 Birbeck, G. L. *et al.* Blantyre Malaria Project Epilepsy Study (BMPES) of neurological outcomes in retinopathy-positive paediatric cerebral malaria survivors: a prospective cohort study. *Lancet Neurol* **9**, 1173-1181, doi:10.1016/S1474-4422(10)70270-2 (2010).

64 Idro, R. *et al.* Severe neurological sequelae and behaviour problems after cerebral malaria in Ugandan children. *BMC Res Notes* **3**, 104, doi:1756-0500-3-104 [pii]
10.1186/1756-0500-3-104 (2010).

65 Opoka, R. O., Bangirana, P., Boivin, M. J., John, C. C. & Byarugaba, J. Seizure activity and neurological sequelae in Ugandan children who have survived an episode of cerebral malaria. *Afr Health Sci* **9**, 75-81 (2009).

66 van Hensbroek, M. B., Palmer, A., Jaffar, S., Schneider, G. & Kwiatkowski, D. Residual neurologic sequelae after childhood cerebral malaria. *The Journal of pediatrics* **131**, 125-129 (1997).

67 Kochar, D. K. *et al.* Cerebral malaria in Indian adults: a prospective study of 441 patients from Bikaner, north-west India. *J Assoc Physicians India* **50**, 234-241 (2002).

68 Bondi, F. S. The incidence and outcome of neurological abnormalities in childhood cerebral malaria: a long-term follow-up of 62 survivors. *Trans R Soc Trop Med Hyg* **86**, 17-19 (1992).

69 Carme, B., Bouquety, J. & Plassart, H. Mortality and sequelae due to cerebral malaria in African children in Brazzaville, Congo. *The American journal of tropical medicine and hygiene* **48**, 216-221 (1993).

70 van Hensbroek, M. B., Palmer, A., Jaffar, S., Schneider, G. & Kwiatkowski, D. Residual neurologic sequelae after childhood cerebral malaria. *J Pediatr* **131**, 125-129 (1997).

71 Wasay, M., Taqi, A., Aziz, H., Azam, I. & Beg, M. A. Neurological involvement in patients with falciparum malaria; frequency and prognostic value. *Clinical neurology and neurosurgery* **113**, 104-106 (2011).

72 Boivin, M. J. Effects of early cerebral malaria on cognitive ability in Senegalese children. *J Dev Behav Pediatr* **23**, 353-364 (2002).

73 Dugbartey, A. T., Spellacy, F. J. & Dugbartey, M. T. Somatosensory discrimination deficits following pediatric cerebral malaria. *Am J Trop Med Hyg* **59**, 393-396 (1998).

74 Kihara, M. *et al.* Impaired everyday memory associated with encephalopathy of severe malaria: the role of seizures and hippocampal damage. *Malar J* **8**, 273, doi:1475-2875-8-273 [pii]

10.1186/1475-2875-8-273 (2009).

75 Carter, J. A. *et al.* Severe falciparum malaria and acquired childhood language disorder. *Dev Med Child Neurol* **48**, 51-57, doi:10.1017/S0012162206000107 (2006).

76 Carter, J. A. *et al.* Developmental impairments following severe falciparum malaria in children. *Trop Med Int Health* **10**, 3-10, doi:10.1111/j.1365-3156.2004.01345.x (2005).

77 John, C. C. *et al.* Cerebral malaria in children is associated with long-term cognitive impairment. *Pediatrics* **122**, e92-99, doi:10.1542/peds.2007-3709 (2008).

78 Boivin, M. J. *et al.* Cognitive impairment after cerebral malaria in children: a prospective study. *Pediatrics* **119**, e360-366, doi:peds.2006-2027 [pii]

10.1542/peds.2006-2027 (2007).

79 Boivin, M. J. *et al.* Developmental outcomes in Malawian children with retinopathy-confirmed cerebral malaria. *Tropical Medicine and International Health* **DOI 10.1111/j.1365-3156.2010.02704.x** (2010).

80 Bangirana, P. *et al.* Severe malarial anemia is associated with long-term neurocognitive impairment. *Clinical Infectious Diseases*, doi: 10.1093/cid/ciu1293-doi: 1010.1093/cid/ciu1293 (2014).

81 Bangirana, P. *et al.* Severe malarial anemia is associated with long-term neurocognitive impairment. *Clinical infectious diseases : an official publication of the Infectious Diseases Society of America* **59**, 336-344, doi:10.1093/cid/ciu293 (2014).

82 Kariuki, S. M., Abubakar, A., Newton, C. R. & Kihara, M. Impairment of executive function in Kenyan children exposed to severe falciparum malaria with neurological involvement. *Malaria journal* **13**, 365, doi:10.1186/1475-2875-13-365 (2014).

83 Holding, P. A., Stevenson, J., Peshu, N. & Marsh, K. Cognitive sequelae of severe malaria with impaired consciousness. *Trans R Soc Trop Med Hyg* **93**, 529-534 (1999).

84 Fernando, D., de Silva, D. & Wickremasinghe, R. Short-term impact of an acute attack of malaria on the cognitive performance of schoolchildren living in a malaria-endemic area of Sri Lanka. *Trans R Soc Trop Med Hyg* **97**, 633-639 (2003).

85 Fernando, D., Wickremasinghe, R., Mendis, K. N. & Wickremasinghe, A. R. Cognitive performance at school entry of children living in malaria-endemic areas of Sri Lanka. *Trans R Soc Trop Med Hyg* **97**, 161-165 (2003).

86 Fernando, S. D. *et al.* The impact of repeated malaria attacks on the school performance of children. *Am J Trop Med Hyg* **69**, 582-588 (2003).

87 Thuilliez, J. *et al.* Malaria and primary education in Mali: A longitudinal study in the village of Donéguébougou. *Social Science & Medicine* **71**, 324-334, doi:http://dx.doi.org/10.1016/j.socscimed.2010.02.027 (2010).

88 Al Serouri, A. W., Grantham-McGregor, S. M., Greenwood, B. & Costello, A. Impact of asymptomatic malaria parasitaemia on cognitive function and school achievement of schoolchildren in the Yemen Republic. *Parasitology* **121 (Pt 4)**, 337-345 (2000).

89 Nankabirwa, J. *et al.* Asymptomatic Plasmodium Infection and Cognition among Primary Schoolchildren in a High Malaria Transmission Setting in Uganda. *The American Journal of Tropical Medicine and Hygiene* **88**, 1102-1108, doi:10.4269/ajtmh.12-0633 (2013).

90 Bangirana, P. *et al.* Reliability of the Luganda version of the Child Behaviour Checklist in measuring behavioural problems after cerebral malaria. *Child Adolesc Psychiatry Ment Health* **3**, 38, doi:10.1186/1753-2000-3-38 (2009).

91 Sowunmi, A. Psychosis after cerebral malaria in children. *J Natl Med Assoc* **85**, 695-696 (1993).

92 Sowunmi, A., Ohaeri, J. & Falade, C. Falciparum malaria presenting as psychosis. *Tropical and geographical medicine* **47**, 218-219 (1994).

93 Pullan, R. L., Smith, J. L., Jasrasaria, R. & Brooker, S. J. Global numbers of infection and disease burden of soil transmitted helminth infections in 2010. *Parasit Vectors* **7**, 37, doi:10.1186/1756-3305-7-37 (2014).

94 Kvalsvig, J. & Albonico, M. Effects of geohelminth infections on neurological development. *Handb Clin Neurol* **114**, 369-379, doi:10.1016/B978-0-444-53490-3.00029-7 (2013).

95 Ezeamama, A. E. *et al.* Treatment for Schistosoma japonicum, reduction of intestinal parasite load, and cognitive test score improvements in school-aged children. *PLoS neglected tropical diseases* **6**, e1634, doi:10.1371/journal.pntd.0001634 (2012).

96 Hotez, P. J. *et al.* The global burden of disease study 2010: interpretation and implications for the neglected tropical diseases. *PLoS neglected tropical diseases* **8**, e2865, doi:10.1371/journal.pntd.0002865 (2014).

97 Coyle, C. M. Schistosomiasis of the nervous system. *Handb Clin Neurol* **114**, 271-281, doi:10.1016/B978-0-444-53490-3.00022-4 (2013).

98 Ferrari, T. C. & Moreira, P. R. Neuroschistosomiasis: clinical symptoms and pathogenesis. *Lancet Neurol* **10**, 853-864, doi:10.1016/S1474-4422(11)70170-3 (2011).

99 Nokes, C. *et al.* Evidence for an improvement in cognitive function following treatment of Schistosoma japonicum infection in Chinese primary schoolchildren. *Am J Trop Med Hyg* **60**, 556-565 (1999).
