# Supplementary material for: Global research priorities for infections that affect the nervous system
Source: Nature. Author manuscript; Available in PMC 2016 May 19. (PMC4697933; doi:10.1038/nature16033)
Supplement: Supplementary material 2 [file NIHMS743819-supplement-Supplementary_material_2.pdf]

Supplemental Table 2. Potential areas for intervention in infectious diseases that affect the nervous system, with references

Disease	Vaccine available	Control of Zoonotic Reservoirs	Control of Vector populations	Treatment
**VIRAL**				
Dengue	New dengue vaccines being tested in large field trials [1]	NA	Yes	None available
Chikungunya	No	NA	Yes	None available
Japanese B encephalitis	Yes	No	Yes	None available
Rabies	Yes	Yes	NA	None available
HSV encephalitis	No	NA	NA	Yes
VZV	Yes	NA	NA	Yes
Congenital CMV	No	NA	NA	Yes
**HIV-RELATED**				
HIV	No	NA	NA	Yes
Cryptococcal meningitis	No	NA	NA	Yes
Toxoplasma	No	Yes	NA	Yes
**BACTERIAL**				
Neonatal sepsis and meningitis	No	NA	NA	Yes
Bacterial meningitis	Yes, for H. influenza type b, and pneumococcal (multiple serotypes) and meningococcal (A, C, Y, W-135) meningitis	NA	NA	Yes
Tuberculous meningitis	Partial protection provided by BCG vaccination [2]	Infrequent (cases due to *M. bovis* and *M. caprae*, which are present in cattle, reported)[3]	NA	Yes
Neurosyphilis				
**PARASITIC**				
Neurocysticercosis	No. Porcine vaccine being tested [4]	Yes	NA	Yes
Malaria	RTS,S vaccine had efficacy in phase 3 studies[5]; other vaccines being developed	NA except for *P. knowlesi*	Yes	Yes
Soil-transmitted helminths	No. Hookworm vaccine in phase I trials but linked to adverse events[6]	NA for the primary soil-transmitted helminths	NA	Yes
Schistosomiasis	No. Phase I vaccine trials [7]	Bovine vaccine trials underway for *S. japonicum* [8]	Yes	Yes

Abbreviations: NA, not applicable

REFERENCES

1 Villar, L. *et al.* Efficacy of a tetravalent dengue vaccine in children in Latin America. *N Engl J Med* **372**, 113-123, doi:10.1056/NEJMoa1411037 (2015).

2 Rodrigues, C. L. *et al.* Spectrum of cognitive impairment in neurocysticercosis: differences according to disease phase. *Neurology* **78**, 861-866, doi:10.1212/WNL.0b013e31824c46d1 (2012).

3 Hansen, N. *et al.* Human Tuberculous Meningitis Caused by Mycobacterium caprae. *Case reports in neurology* **4**, 54-60, doi:10.1159/000337299 (2012).

4 Lightowlers, M. W. Control of Taenia solium taeniasis/cysticercosis: past practices and new possibilities. *Parasitology* **140**, 1566-1577, doi:10.1017/S0031182013001005 (2013).

5 Rts, S. C. T. P. *et al.* A phase 3 trial of RTS,S/AS01 malaria vaccine in African infants. *N Engl J Med* **367**, 2284-2295, doi:10.1056/NEJMoa1208394 (2012).

6 Diemert, D. J. *et al.* Generalized urticaria induced by the Na-ASP-2 hookworm vaccine: implications for the development of vaccines against helminths. *The Journal of allergy and clinical immunology* **130**, 169-176 e166, doi:10.1016/j.jaci.2012.04.027 (2012).

7 Riveau, G. *et al.* Safety and immunogenicity of rSh28GST antigen in humans: phase 1 randomized clinical study of a vaccine candidate against urinary schistosomiasis. *PLoS neglected tropical diseases* **6**, e1704, doi:10.1371/journal.pntd.0001704 (2012).

8 Shi, F. *et al.* Field testing of Schistosoma japonicum DNA vaccines in cattle in China. *Vaccine* **20**, 3629-3631 (2002).
